# Smart Airport Cybersecurity: Threat Mitigation and Cyber Resilience Controls †

**DOI:** 10.3390/s19010019

**Published:** 2018-12-21

**Authors:** Georgia Lykou, Argiro Anagnostopoulou, Dimitris Gritzalis

**Affiliations:** Department of Informatics, Athens University of Economics & Business (AUEB), GR-10434 Athens, Greece; lykoug@aueb.gr (G.L.); anagnostopouloua@aueb.gr (A.A.)

**Keywords:** smart airports, cyber-security, cyber-resilience, internet of things, critical infrastructure protection

## Abstract

Airports are at the forefront of technological innovation, mainly due to the fact that the number of air travel passengers is exponentially increasing every year. As a result, airports enhance their infrastructure intelligence and evolve as smart facilities to support growth, by offering an enjoyable travel experience. New challenges are coming up, which aviation has to deal with and adapt to, such as the integration of Industrial IoT (Internet of Things) in airport facilities and the increased use of smart devices from travelers and employees. Cybersecurity is becoming a key enabler for safety, which is paramount in the aviation context. Smart airports strive to provide optimal services in a reliable and sustainable manner, by working around the domains of growth, efficiency, safety and security. This article researches: (a) the implementation rate of cybersecurity measures in commercial airports; (b) malicious threats that evolve due to IoT and smart devices installed; (c) risk scenario analysis for IoT malicious attacks with threat mitigation actions. With the aim to enhance operational practices and develop robust cybersecurity governance in smart airports, we present a systematic and comprehensive analysis of malicious attacks in smart airports, to facilitate airport community comprehend risks and proactively act, by implementing cybersecurity best practices and resilience measures.

## 1. Introduction

Airport operations and business models have evolved dramatically over the last decades to support the explosive growth of the global aviation industry [1]. Regulatory reform in the new air travelling era produced dramatic traffic growth, diversity and choice for airline passengers. As airlines refine their operating models to align growth to efficiency, airports evolve in parallel to create massive networks of hubs and intelligent systems, which together create an efficient air transportation ecosystem [2]. Since airports are considered a gateway to the world for travelers and business, they are of great importance for country development and economic growth [3]. This article is an extended version of an earlier conference paper with title: “Implementing Cyber-Security Measures in Airports to Improve Cyber-Resilience” [4].

In the USA, aviation and airports, as a transportation subsector, constitute a critical infrastructure and key resource sector, according to the U.S Homeland Security Presidential Directive [5]. The same applies in Europe, where critical infrastructures and essential services in air transport facilities should be adequately protected according to NIS (Network and Information Systems) directive, EPCIP (European Programme for Critical Infrastructure Protection) and European Community EC/216/2008 regulation [6,7,8]. 

Securing smart airports and staying ahead of evolving cyber threats is a shared responsibility, involving airlines, airports, vendors and regulators [7]. Identification of cyber-threats challenges, risk assessment approaches and guidelines to enhance cyber security are priorities currently researched by the aviation industry. This article extends previous work [4] aiming to identify cybersecurity policies in airports operating intelligent services, while taking stock of measures and good practices already implemented. 

In this work, malicious cyber-threats that may influence the operational efficiency of smart airports, when equipped with IoT applications, are developed and analyzed. We present an overview of malicious risks that can affect essential services in airports and interconnected networks. We also illustrate a series of analytical malicious attacks scenarios in critical airport’s infrastructures, along with mitigation strategies and resilience measures. The contributions of this paper are the following: (i) a research analysis of measures and best practices currently implemented to commercial airports, analyzed based on online survey data; (ii) a detailed identification of malicious threats for IoT applications in smart airports; (iii) scenario analysis of malicious attacks in smart airports assets, including cascading effects, mitigation actions and cyber-resilience measures.

The remainder of this article is structured as follows: Section 2 describes research methodology, while theoretical framework is presented in Section 3, including airports’ classification based on their technological evolution. Online survey and research results are analyzed in Section 4. Malicious threats analysis and detailed attack scenarios are presented in Section 5. Malicious attacks motives are also discussed and attributed to each attack scenario. Finally, in Section 6 research conclusions are presented.

## 2. Research Methodology

This work has been developed using a combination of literature research and information received from an online survey about airport cybersecurity. The survey was addressed to European and American busiest airports with the purpose to understand the opinion of airport IT personnel about the introduction of the IoT to their airports and the cybersecurity measures applied. The format of this survey and other details, including questionnaire, is provided in the Appendix A. All survey responses received were promised to be treated with confidentiality and data from this research is reported only in the aggregate. 

Our research goal was to define the implementation rate of cyber-security best practices in combination with IoT application status, through IT personnel opinions. Since there was a great diversity of technological evolution in airports examined, we have made an aggregated analysis of responses, combined with airport classification presented in Section 3.1. Based on survey results, we extended our research, in order to develop threat scenario analysis for malicious cyber-attacks that may influence the operational efficiency of smart airports.

## 3. Theoretical Framework

Cyber security can be defined as the collection of tools, policies, security safeguards, guidelines, risk management approaches, training, best practices, assurance and technologies used to protect the cyber environment and organizations’ assets [9]. Although many airports have robust systems in place to address common hacking threats, they haven’t always taken a holistic approach to the IT cyber environment or considered the broader threat to the aviation system. In this direction, International Civil Aviation (ICAO) with ICAO/A39 calls on states and industry stakeholders to encourage coordination with regard to aviation cybersecurity strategies, policies and sharing of information to identify critical vulnerabilities that need to be addressed, by developing systematic information sharing on cyber threats, incidents and mitigation efforts [10]. Following this direction, a variety of standards has been developed such as: (i) European Norm (EN) 16495 standard for Air Traffic Management tailored to civil aviation with supporting guidance on Information security for organizations, supporting civil aviation operations; (ii) International Society of Automation (ISA)/International Electrotechnical Commission (IEC)-62443 which is a set of standards, technical reports, and related information that define procedures for implementing electronically secure Industrial Automation and Control Systems; (iii) National Institute of Standards and Technology (NIST)-Special Publication 800-53 about Security and privacy controls for Federal information systems and organizations, which is a comprehensive catalogue of controls with much supporting advice; (iv) NIST 800-82 Guide to Industrial Control Systems (ICS) Security, which provides guidance through typical system topologies, threats and vulnerabilities.

The aviation sector and especially smart airports cybersecurity have attracted researchers in the recent years, as the incorporation of new innovative technologies and their available attack surface has been increased [1,2,3,7,8,9,10,11,12,13]. Civil Air Navigation Services Organization (CANSO) [14] developed a guide for increasing security level to Air Traffic Management (ATM), by presenting cyber threats and risks, as well as threat actors with their motives. CANSO proposed a model in order cyber security to be addressed, in combination with international standards, NIST Cybersecurity Framework, as well as a risk assessment methodology.

Although significant research has been presented regarding ATM cyber risks, there is a lack of research about threats and vulnerabilities for ground handling IT systems and airport services, especially when equipped with smart applications. Particular to airport cyber security, risks constantly change, as new threats and vulnerabilities evolve, along with ever-changing technology implementations. In 2013, Gopalakrishnan et al. [2] made an analysis about cyber-security in airports, giving a roadmap to secure control systems in the transportation sector, by presenting cyber risks in airport operations and potential targets for cyber-attacks. Existing vulnerabilities in Airport ICS have been evaluated by US Airport Cooperative Research Program and a Guidebook on Best Practices for Airport Cybersecurity has been published in 2015, to mitigate inherent risks of cyberattacks on technology-based systems [11]. The European Union Agency for Network and Information Security (ENISA) has published its continuing work [15,16,17,18] on communication network dependencies in industrial infrastructures, focusing on ICS/SCADA (Supervisory Control and Data Acquisition) systems and IoT infrastructures. In 2016, ENISA also published a security guidance for smart airports [7], presenting key stakeholders, asset groups, threats and risk analysis, best practices and security recommendations addressed to airport decision makers, policy-makers and industry stakeholders. Suciu et al. [19] presented use cases of attacks in airports and explained which prevention methodology can be implemented, in order to improve the security level with the integration of several security tools, services and fields. Afify et al. [20] focused on analyzing Denial of Service (DoS) attacks that occur in airports and especially in their automation systems by describing how attacks are launched along with effective countermeasures. Moreover, U.S. Department of Homeland Security [21] published a report which analyzed botnets and other automated, distributed threats, pointing out that such types of attack are a global problem nowadays. Finally, SESAR research addressed cybersecurity issues in Airport Operations Centers including a comprehensive maturity model to approach to cyber-security within European ATM and to develop a comprehensive response to cyber-threats [22]. 

Although threats to smart airport’s cyber security apply to broad categories of assets (such as communication networks, servers and control systems, internal/sensitive information, authentication and access control systems), the majority of researchers focus on one or two scenarios of attack, while addressing cybersecurity issues in airports. To the best of our knowledge, no one has presented a complete scenario analysis of malicious attacks that may happen in smart airports, concerning IoT technologies and smart applications, including mitigation actions, resilience measures and impact effects on the information security triad (Confidentiality-Integrity-Availability: CIA). 

### 3.1. Airport Intelligence Classification

As airports have enhanced their interoperability capabilities, by using IοT technology and intelligent applications, to achieve on effectiveness, infrastructure complexity has been enormously increased. According to Pethuru [1], there is an evolution pace in today’s airports, which can be classified into three broad categories: (i) Basic Airports, which focus on capabilities necessary for safe and efficient management of landings, departures, and other aircraft operations, by offering basic passenger services; (ii) Agile Airports, which adapt to this changing digital environment, by offering shared services on a common platform; and (iii) Smart Airports, which fully exploit the power of emerging and maturing technologies of IoT, with advanced and pervasively deployed sense-analyze-respond capabilities. By enabling the exchange of real-time information, profound collaboration, and airport-wide process integration, smart airports significantly improve operational efficiencies, passenger services, and advanced security capabilities. 

According to ENISA, smart airports are those who make use of networked, data driven response capabilities that, on the one hand, provide travelers with a better travel experience and on the other hand, aim to guarantee higher levels of security for the safety of passengers, operators and general public [7]. Since, safety and security are the most significant domains in the aviation context, a safe environment must be ensured by proactively handling difficult cyber challenges, while minimizing operations disruption. In this work, we have concentrated our research on mapping smart airport’s threats, initiated from malicious actions, in order to develop a variety of attack scenario analysis, along with recommended mitigations and resilience measures.

## 4. Online Survey Results 

**Figure 1:**
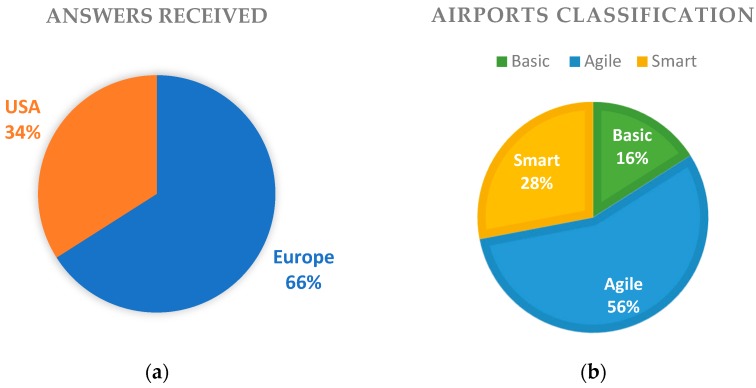
(**a**) Source of airports’ replies; (**b**) Airports’ classification based on IoT apps.

An online survey questionnaire was addressed to the 200 busiest commercial airports in Europe and USA, although only one third of them had responded to the survey. Among them, we distinguished fully completed and solid questionnaires and we elaborated their results. Answers received from European airports reached 66%, while 34% came from USA as shown in Figure 1a. The airports have been further classified to Basic/Agile/Smart categories, according to their own statement about being or planning to be smart, in combination with the number of IoT applications that they have indicated to use in their facilities. This classification was chosen, in order to better evaluate the cyber-security preparedness level of airports, based on ICT complexity and technological progress. As a result, 16% of airports have been classified in the basic category, 56% were categorized as agile and the rest 28% of airports were ranked as smart airports, as presented in Figure 1b.

Although 59% of responders stated having effective cybersecurity policies for IT assets, when they were asked to rank the risks from IoT devices, the majority (76%) pinpointed the lack of security awareness as the greatest risk, followed by internet connectivity risk (29%), which reveals a controversy in security confidence of responders.

**Figure 2:**
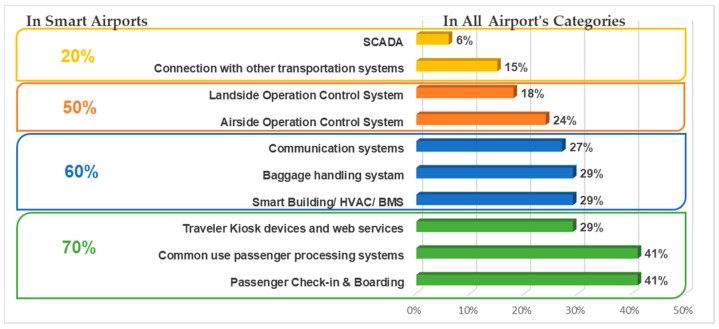
IoT applications in airports.

Airports have defined which smart applications are using in their facilities that underpin key airport activities, as listed in Figure 2. The percentages, listed on the right side of Figure 2, are presenting the overall performance from all airport’s answers received, while on the left side, the performance of smart airports is exhibited. As we can see, the most popular smart applications, used in all airports, are passenger check in and boarding services (41%), common use passenger processing systems (41%), while the least used are SCADA applications (6%) and connections with other transport systems (15%). 

Especially in smart airports, IoT applications like baggage handling, passenger check in, landside operation controls, common use passenger services and traveler web services are found to be used in frequencies over 70%. Building Management Systems (BMS) and HVAC (Heating, Ventilation, Air Conditioning) equipment controls are also widely used in smart airports (60%). SCADA systems are in their infancy stage overall in airports. Only smart airports have stated to use SCADA with a 20% implementation rate.

Since industrial IoT applications are in their emergence, a great expand is expected to transform mainstream busiest airports to smart airports. Research revealed that this early adoption increases smart airport’s interoperability, along with vulnerability exposure to cyber threats [2,7].

Security good practices and tools have been developed and published in literature [5,6,7,13]. The identified practices for smart airports have been categorized into three main groups: (i) Technical good practices; (ii) Organizational good practices; and (iii) Policies and Standards. 

**Figure 3:**
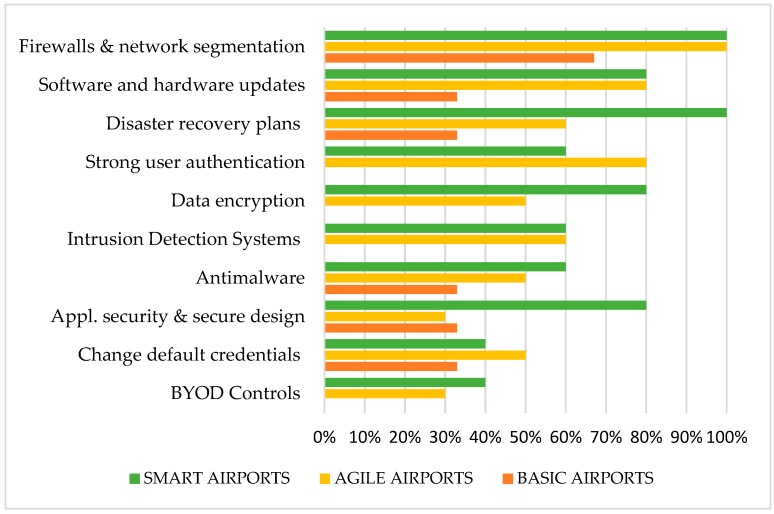
Technical good practices implementation analysis.

Figure 3 exhibits the technical good practices implemented in all airport’s categories, based on survey answers and airport classification. An analytical description of these measures, along with survey responses per airport category, have been presented in our previous work [4]. As we can observe, the most implemented technical based practices for all airports are: (i) Firewalls and network segmentation; (ii) Software and hardware updates; and (iii) Disaster recovery plans. On the contrary, the least implemented technical based practices are: (i) BYOD: Bring Your Own Device Controls; (ii) Change default credentials; and (iii) Application security and secure design. 

Smart airports have the greatest implementation rate of technical practices, reaching 70% on average, since advanced complexity of smart applications, requires advanced cybersecurity defense. However, we have found that some practices were poorly implemented by smart airports, such as changing default credentials and BYOD controls, which reveals a security gap and possible areas for cybersecurity amelioration.

Agile airports have an overall lower implementation rate of technical practices, reaching on average 59%. They are all implementing firewalls and network segmentation, while the majority uses strong authentication and software/hardware updates. However, they lack of applying technical practices, like BYOD Controls and secure application design.

Basic airports need to start implementing technical measures like: Data encryption; Strong user authentication; BYOD controls and IDS, since they have responded not to apply at all. The most implemented measures are firewalls and network segmentation at 67% rate, while the average implementation on technical practices is only 23%.

Research also revealed that airports, who are using IoT and SCADA applications in their facilities, have more technical practices implemented than the other airports. This indicates a higher concern about cybersecurity and effective performance towards cyber resilience achievement.

**Figure 4:**
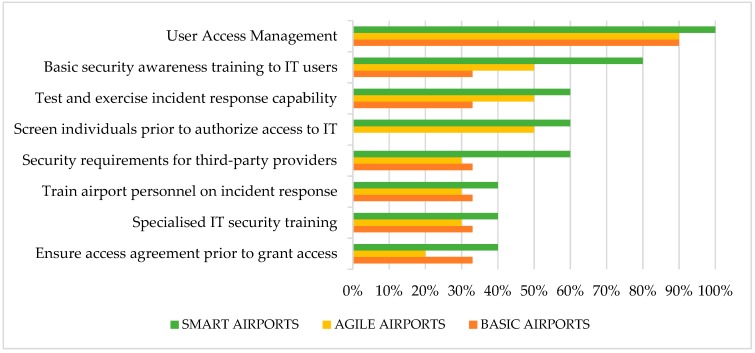
Good practices about airport’s organization and processes.

Figure 4 summarizes employees’ responses about airport organizational practices, applied to all airport’s categories. An analytical description of these measures, along with survey responses per airport category, have been analyzed in our previous work [4]. The most implemented security process is user access, reaching 95% overall performance, followed by basic security awareness training to all information system users. The least implemented practices are: (i) access agreement for third party stakeholders; (ii) specialized security training; and (iii) training on incident response for airport’s personnel.

Smart airports have a good implementation rate of organizational practices reaching 60%, agile airports have on average low implementation rate reaching 44% and basic airports implement them with the disappointing performance of 36%. Moreover, basic airports seem to ignore basic policies and keep low performance to the majority of organizational practices. This can seriously impact their cyber resilience capability, in case of a cybersecurity incident in critical IT processes and services. 

**Figure 5:**
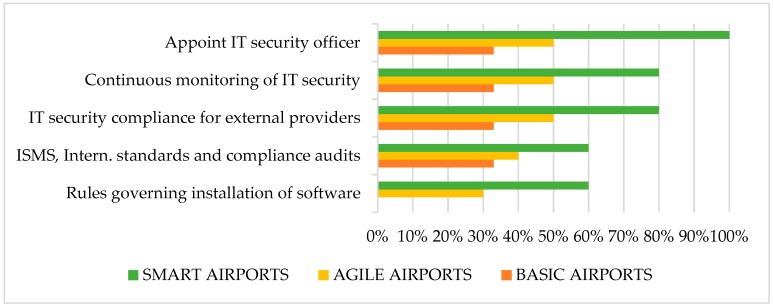
Good practices for policies and standards.

A variety of airport security policies and standards exists in literature [7,13]. Figure 5 summarizes IT personnel responses about airport policies and standards applied, according to airport classification and overall. An analytical description of these measures, along with survey responses per airport category, have been analyzed in our previous work [4]. The most implemented security policies are the appointment of IT security officer, continuous monitoring of security and information security compliance from providers of external IT services. Unexpectedly, the least implemented policy is to enforce rules governing installation of software. This is essential to enhance cybersecurity efficiency, in view of the increase of personal devices interacting with airport’s IT systems, combined with the lack of BYOD security controls.

## 5. Smart Airports Attacks: Scenario Development

### 5.1. Cybersecurity Malicious Threats Analysis

**Figure 6:**
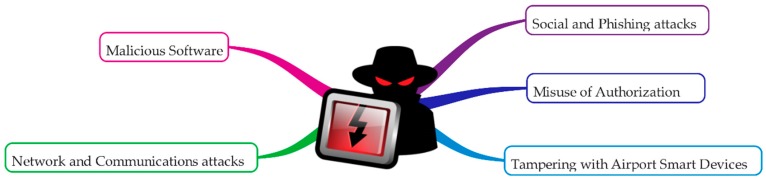
Cybersecurity malicious threats categories.

Although many security threats may occur either by intentional or unintentional factors, in this work we have focused on cyber security threats that spring from intentional /malicious actions. Various methods can be used by actors with malicious intent to compromise IT assets or to perform elevation of privilege attacks. Each of these attacks may lead to security incidents with breach of confidentiality, integrity, availability and should be considered while assessing the attack vectors for each asset, in order to protect the airport’s safety and business continuity. Major cybersecurity threats against IoT applications in smart airports can be segregated into the following categories: (i) Network and communication attacks; (ii) Malicious software; (iii) Tampering with Airport Smart Devices; (iv) Misuse of Authorization; and (v) Social and Phishing attacks. Figure 6 presents these malicious threat categories, which are further analysed afterwards.

(1) Network and Communication attacks: Networks are subject to attacks from malicious sources, divided into two categories: passive attacks, where intruder intercepts data and active attacks, where actor disrupts the network’s normal operation and gains access to assets available via this network. Despite the legislation, which prevents communication interception, smart airports remain an attractive target of tampering or network attacks, depending on the attack surface and controls in place. Various kinds of wireless communications may be affected or jammed, such as wireless communications, air traffic management and radio signals, which they can be overshadowed by jamming devices. Denial of Service attacks also enable attackers to disrupt information systems and networks, being able to impact on airport’s system availability. As a result, network outages, passenger delays, cancelled flights may have serious imparts to smart airports, along with loss of confidence, damages to reputation, and potential financial damages.
(2) Malicious software: Malware, which is able to infect common information systems, may also compromise smart devices, including passenger and staff portable devices, servers and other smart components, including airport’s supervisory control and data acquisition systems. Severe impact on airport’s infrastructure occurs, since such software acts maliciously, misusing its ambient authority on the computer it manages to get installed and runs on. Vulnerabilities may exist in smart airport systems, including third party security issues on smart assets, remote sensors and controls. Any smart airport system with an available attack surface, where security fix has not applied and system is not running with all the latest security patches is a likely target of malicious software attack [7].
(3) Tampering with Airport Smart Devices: Airport devices can be tampered with various unauthorized ways. Unauthorized modification includes manipulation of data at central reservation systems, administration IT systems, airport’s stored sensor data. The threat of tampering also includes unauthorized modification of hardware or software with data deletion or corruption, which can affect the behavior of airport’s self-serving systems like automatic check in machines, passport control gates and smart building management systems. As a result, attackers can potentially gain control over systems, and result in malicious behavior with physical safety breaches and serious impact on airport’s security.
(4) Misuse of Authorization: Although, access controls are security features which define how users and systems interact, attackers may be able to obtain credentials and escalate authorization rights. Even employees or contractors, acting as insider threat and possessing authorization rights may be able to misuse their privileges. Such attacks also include credential theft via social engineering, spear phishing or simply insider threats. Provided that attackers can gain access, holding legitimate user’s credentials, they can also escalate their privileges, and damage smart airport assets, depending on the level of privilege obtained.
(5) Social and Phishing attacks: Social engineering can manipulate or mislead people in order to perform actions on behalf of the attacker. Social attacks are effective as they can circumvent technical and physical controls. Airport employees who lack security awareness and may not follow procedures, can pose a significant risk to airport cyber security. Email remains a primary method for threat actors to infiltrate a system, enabling the attackers to gain full access to the victims’ accounts, identity and authorization. Even though organizations install filtering capabilities, phishing emails still may get through and trick the victim to perform a malicious action without knowing.

### 5.2. Attack Scenarios Analysis Along with Mitigation and Resilience Measures

In our research we have evaluated various attack scenarios relevant to Smart airports and IoT applications and created a collection of scenario attacks for all malicious threats previously introduced. A more detailed description for each scenario is presented, along with domain and impacted assets, as well as possible escalation to cascading effects on other critical assets. Additionally, a step attack with graphical representation depicts malicious attack phases and related impact on security parameters. In addition, wherever examples of real incidents of cyber-attacks in smart airports have been investigated, are also referenced. Finally, mitigation actions and resilience measures that could be deployed in each scenario are presented, in order airport’s cybersecurity and cyber-resilience to be enhanced against malicious attacks.

#### 5.2.1. Malicious Attack: Distributed Denial of Service attack

**Figure 7:**
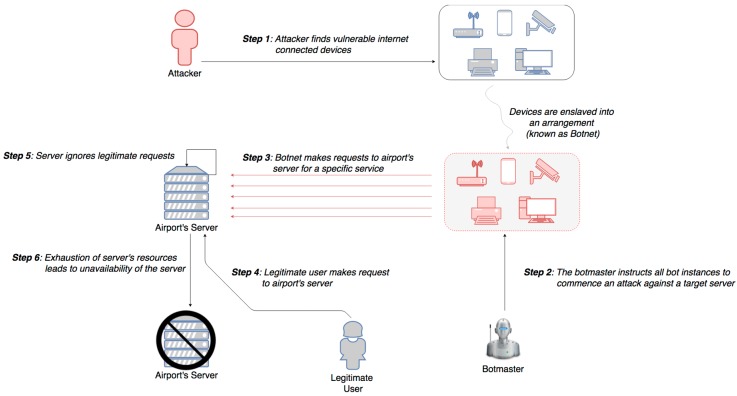
DDoS attack using Botnets.

The main characteristic of smart airports is the networked, data-driven response capabilities through smart components and integrated IoT devices. Any smart device connected to airport’s network may support crucial key functions of interoperability between aircrafts, airport administration, air traffic control, and other forms of communication. Distributed Denial of Service (DDoS) attack is one of the most interesting and widely seen cyber-attack in the recent times, as presented in Figure 7. In DDoS attack, a hacker temporarily enslaves a number of internet-enabled devices into an arrangement and then make simultaneous requests to a server or an array of servers for a specific service, thereby overwhelming the server and make it ignore legitimate requests from end-users. The Mirai Botnet code is an example of DDoS attack, which infects poorly protected internet devices, to find those that are still using their factory default username and password [23]. The effectiveness of Mirai is due to its ability to infect thousands of these insecure devices and co-ordinate them to mount a DDoS attack against a chosen victim. Successful DDoS attack can result in either access deny for the legitimate users or system’s inability to distinguish legitimate users from fake ones. 

Impact Evaluation: Launch of a DDoS attack impacts the availability of smart airport’s resources and services. The consequences are varied according to the time needed for recovery of business operations. This type of attack may lead to actions, like cancelation of flights, passenger delays, unavailability of cloud-based services, or even outage of staff communication systems. Such an attack happened in June 2015, at Warsaw Chopin airport, where approximately 1,400 passengers were grounded for five hours, while Polish airline was the victim of a DDoS attack [24]. 

Cascading effects: Once a smart airport’s network is flooded and non-responsive to requests, many of its functionalities become unavailable. Some of the consequences of such unavailability may refer to the proper operation of passenger management systems, including kiosk devices or passenger check-in and boarding. Moreover, the domains of safety and security, as well as the Airline/Airside Operations may be vulnerable to this attack, as their operations are mainly based on the airport’s network. Last but not least, the asset group of IT and Communications may probably be affected as it contains internet-connected assets. Indicative examples of these assets are cloud-based data and application services, passenger-airline communication systems, as well as common communication systems.

Mitigation Actions: Security hardening of airport’s smart devices and IoT systems is a quite important mitigation action, as there is need for the reduction of the existing attack surface. This action can be achieved by changing default passwords, disabling services, closing ports, as well as regular patching of systems. Smart airport’s network should be protected by firewalls and follow a defense-in-depth approach, in order the traffic between the network segments and hosts to be more restricted. Moreover, another way to protect against DDoS attacks is through volumetric protection from the Internet Service Provider (ISP). Most ISPs have the ability to automatically detect potential DDoS attacks and to filter/throttle back requests from possible sources. The ISP is then able to identify and mitigate abnormal traffic to only deliver ‘normal’ requests to the final IP address [22]. An alternative method of defense could be to have a secondary Internet connection and another IP range, so as to be used in a case of emergency, in order to secure airport operations.

Resilience Measures: In order smart airports to react immediately to a potential DDoS attack, involves the combination of attack detection, classification and response tools, aiming to block illegitimate traffic. Therefore, it is important to regularly exercise preparedness and response time on test incidents, as well as provide incident response capabilities. In addition, communication of anomalous activity and malicious attacks to IT staff, senior management, affected stakeholders, other agencies, and law enforcement personnel can help the airport community to be better prepared and defend against similar attacks.

#### 5.2.2. Malicious Attack: Communication Attack to ATM Systems

Automatic Dependent Surveillance–Broadcast (ADS–B) is a recently introduced surveillance technology, where aircraft’s navigation system determines its position, using a separate global positioning source (GPS), and periodically broadcasts it together with other data, such as aircraft identity and barometric altitude, enabling it to be seen by any adequately equipped agent [25]. The technology has proven particularly attractive in locations, where previously no form of surveillance was physically possible or economically feasible. According to ICAO, ADS-B is now on track to replace conventional radar surveillance systems and become the backbone of next-generation ATM systems [26].

**Figure 8:**
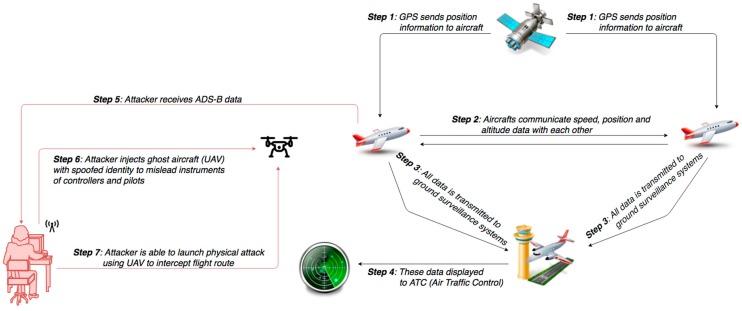
Communication attack on ATM systems.

ADS-B avionics broadcast unencrypted, error-code protected messages over radio transmission links, approximately once per second, containing the aircraft’s position, velocity, identification, and other ATM-related information [27]. Since ADS-B signals are unauthenticated and unencrypted, “spoofing” or inserting a fake aircraft into the ADS-B system can be easily accomplished, as shown in Figure 8. Therefore, the system is susceptible to hacking, where attacks may range from passive actions (eavesdropping) to active attacks. The attacks can be implemented using Universal Software Radio Peripheral, a widely available Software-Defined Radio supported by an ADS-B receiver/transmitter chain with GNU Radio [28]. Along with other researchers, Santamatra has recently published a white paper, presenting active attack scenarios that could result from the weak security posture of satellite communications, revealing how hundreds of in-flight aircrafts are accessible and vulnerable to message jamming, replaying of injection and other active attacks [29].

Impact Evaluation: With open broadcast data and no encryption there is no confidentiality protection for ADS-B communications. Lack of any authentication provides no integrity and the ability to jam signals brings into question availability. As a result, all security parameters may be impacted during such an incident. With air traffic services compromised, only reduced traffic capacity can ensure minimum safety standards. Such an incident happened in September 2017, at Sydney Airport and drove ATM system software failure at Sydney’s Air Traffic Control [30].

Cascading effects: The most fundamental security issue with ADS-B is the core idea of broadcasting the identity and precise location of each aircraft, which could open the door for a terrorist to physically attack an aircraft either by using an Unmanned Aerial Vehicle (UAV) to intercept the flight route, or even by using a missile launcher to target aircraft of a specific airline or corporation. This has already happened in the past, like the incident in 2014, with a flight passing over eastern Ukraine, killing all 283 passengers and 15 crew on board [31]. 

Mitigation Actions: The use of confidentiality and authenticity features in data traffic becomes mandatory. Therefore, cryptographic protection for ADS-B can be an effective mitigation action. It is also important for ground services to have any received data validated, by using legacy surveillance systems, such as primary radar information. Also, in ATM system’s dependability is based on redundancies to ensure efficiency, reliability and continuity of operations. Therefore, Air Traffic Control (ATC) has to maintain in use current network of primary and secondary radars, as backup systems to ADS-B advanced technology. In addition, with the use of Wide Area Multilateration technology (WAM), data that show up on a controller’s screen are multi-purpose validated by surveillance systems, so that spoofed targets are filtered out [22].

Resilience Measures: It is important to regularly exercise ATC stuff and systems preparedness, response time and provide incident response capabilities. An effective contingency plan should be developed, implemented and tested, so that ATM resilience to be improved. Finally, communication of anomalous activity and malicious attacks to Air Traffic Safety Electronics Personnel and law enforcement actions will help the airport to be better prepared and defend against similar attacks.

#### 5.2.3. Malicious Attack: Malicious Software on an Airport’s Network

**Figure 9:**
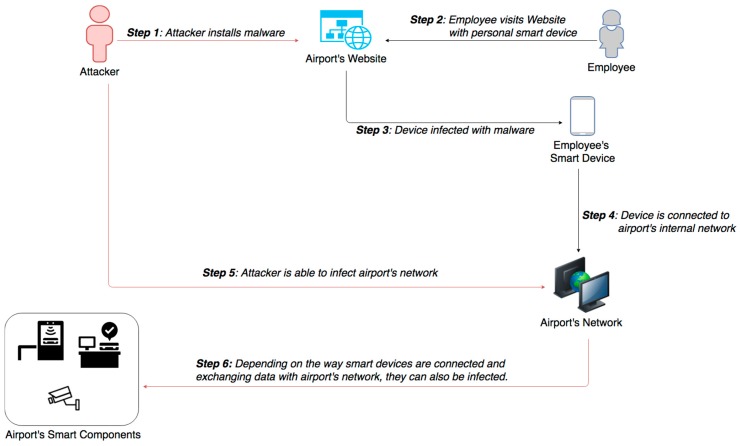
Malicious Software Installation.

Smart airports have complex wired campus networks that allow data access through secondary and tertiary levels of distribution. Having incorporated artificial intelligence systems to their daily functions and by allowing employees and maintenance personnel to use their owned smart devices (BYOD), they became more vulnerable to network malicious attacks. Moreover, aviation and ATM systems have increased the use of IP connections to enhance efficiency and interoperability, which may lead to unauthorized individuals gaining access [19]. Research experiments have shown that any malicious passenger or employee, equipped with a smart device infected with malware, may be able to access the aircraft’s system and even influence system’s integrity [27]. Similar attacks can be accomplished with malware installation to the airport’s website or intranet, where airport users’ devices may be infected, thus giving the opportunity to malicious attackers to access airport’s network and critical information system through these infected devices, as presented in Figure 9. Such an attack happened in September 2016, at Vienna Airport, where computer servers and employee’s computers were infected with malware [32].

Impact Evaluation: Assuming that BYOD can be inserted in both conventional network and the restricted one of internal systems communication, in order to exchange information, a compromised device can impact airport systems integrity and availability. Since airports systems are interconnected to increase their interoperability, potential attacks on airport’s network can cause network outage, flight cancellations, passenger delays, loss of confidence and financial damages. In the unlikely event that aircraft’s avionics systems are also infected, attacker may be able to manipulate data from essential functionalities and services, which can jeopardize airplane safety parameters [11]. While this attack can impact the availability and integrity of information and airport’s resources, the impact on confidentiality has a lesser effect on the smooth operation of airport facilities. This does not mean that we should not bear in mind this impact and therefore implement appropriate countermeasures.

Cascading effects: Once an attacker gains access to airport network or even aircraft avionics systems, he may be able to control other components or applications connected to this system. Indicative examples are air navigation and air traffic control management systems, communications, aircraft collision avoidance systems and other aircraft management systems. Compromising either airport’s or aircraft systems puts at serious risk essential services and facilitates further attacks that may lead to fatal accident and loss of human lives.

Mitigation Actions: For the adequate protection of smart airports, some of the basic countermeasures and BYOD controls, including antimalware and Intrusion Detection & Protection Systems (IDS/IPS), should be implemented. Moreover, smart airports should establish technical controls and organizational policies, in order to protect the infrastructure from potential risks coming from the employees’ personal devices. In addition, strict control measures and severe access restrictions for BYOD on airport’s critical systems or SCADA systems should be applied. Personnel security training and awareness is also vital for the ones, who are allowed to bring and connect their own devices, such as smartphones or tablets, to airport’s systems. Finally, it is quite important that all the airport systems are designed and developed according to international security standards and best practices that drive at organization’s sufficient security. 

Resilience Measures: It is important all the software patches and hardware updates to be done on time, so as smart airport’s systems to be kept up-to-date, with reduced exposure to common vulnerabilities. Moreover, the monitoring and audit of systems and log files are also crucial for the resilience of smart airports, because any unauthorized changes by malicious insiders should be immediately detected. IT stuff should always be efficiently trained and prepared to isolate inffected systems, remove malicious software, recover from new attack vectors, and gain experience from lessons learned.

#### 5.2.4. Malicious Attack: Tampering with Airport Self-Serving Systems

**Figure 10:**
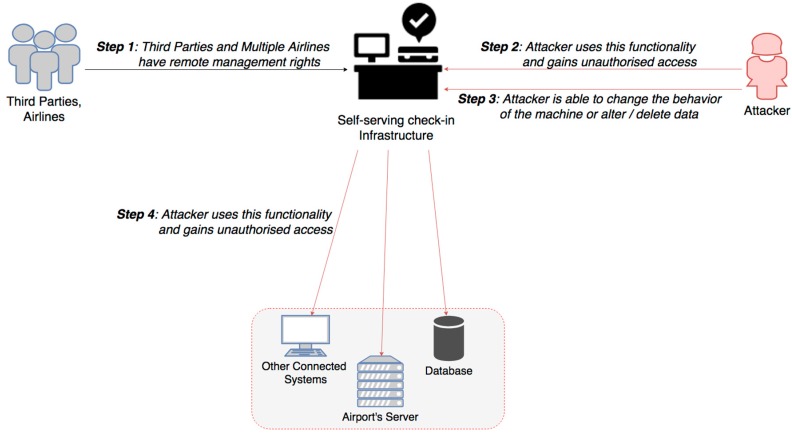
Tampering with airport self-serving systems.

Airline companies foster the use of Common Use Passenger Processing System (CUPPS) to facilitate 24 h/7 days a week customer support and speed up check-in and passenger control processes via automated smart devices. Self-serving check-in infrastructures are being installed nowadays, being used and shared by multiple airlines, along with third parties which also have started to operate common services [7]. The majority of these devices run commonly used operating systems, firmware or proprietary software. Although these devices leverage intranet connectivity for accessing only content to company servers, they often provide remote management functionalities and they may be subject to tampering attacks, as they are exposed in public spaces. An attack scenario is exhibited in Figure 10. Such attacks can also affect various airport systems and SCADA equipment, from baggage handling and access control to air-conditioning and power distribution systems, which are widely distributed across airport infrastructures. While in the past these systems were air gapped, nowadays are networked and interdependent. Thus, smart airports are more likely to be victim of tampering attack, due to their adoption of IoT technologies. Los Angeles Airport has experienced a number of cyber incidents in the past, related to malware that targeted networked baggage systems [2].

Impact Evaluation: Successful tampering can result in the attacker having unauthorized access to the machine and potentially lead to privilege escalation. This enables attacker to change the behavior of the machine, both in terms of the customer facing actions and the interactions with other connected systems. Such an attack happened at Iran’s Mashhad Airport, in May 2018, where hackers took control of the airport’s monitors, in order to express their support to Iran Protests [33]. Disruption of CUPPS or SCADA systems may create inconvenience to passengers and impact airport’s services availability and operational efficiency, such as creating flight cancellations, long passenger waiting queues and longer boarding times [7]. It can also impact integrity of information, for example facilitate the boarding of unknown passengers into the plane and lead to more serious security risks involving safety. Last but not least, tampering on airport devices may impact privacy and data protection, where privilege escalation can lead to loss of personal or sensitive passenger’s data, such as passport, identity or credit card details. 

Cascading effects: Once an airport CUPPS or SCADA devices are compromised, the attacker may be able to infect other interconnected systems and related databases. The lack of logging and forensic capabilities of such systems prevents early diagnosis and response, undermining trust on system’s provided services [22]. Except from any interruption of the provided service operation, a threat that arises here is the alteration of data, aiming at whatever act can compromise the safety of passengers, including potential terrorist facilitation. Although the majority of airports use segregated networks, depending on the effectiveness of the controls, cascading effects have the potential to impact the secure operation of Airside and Landside operations.

Mitigation Actions: The most important mitigation action is to restrict usage of external media drives or wireless connections, disable unused services, so as to minimize equipment communication ports. Also, it is vital to provide along with network segmentation, the adequate physical security. Users shall ensure that unattended equipment has appropriate protection either with security guards, video monitoring or surveillance systems. Moreover, data encryption can minimize privacy risks, while Intrusion Detection & Prevention Systems (IDS/IPS) can support early detection and prevent further impacts on airport operational systems.

Resilience Measures: It is important to provide backup systems, which can be activated to support business continuity in case of such an incident. Basic security awareness training to all airport employees could prevent unauthorized access to these devices, while incident response capacity building for ground staff should be introduced and regularly tested. Finally, an effective contingency plan should be developed, implemented and tested to improve airports operational resilience. 

#### 5.2.5. Malicious Attack: Network attack to CCTV systems

**Figure 11:**
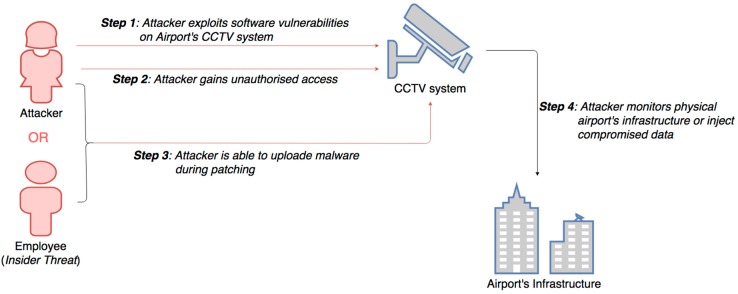
Network attack on CCTV systems.

Digital surveillance systems are integrated nowadays with new ways to speed up airport processes and detect threats, including radio frequency identification (RFID) tags for tracking purposes, new thermal imaging scanning devices, intelligent closed-circuit television (CCTV) programs that identify unusual behavior and devices to detect chemical substances [34]. Digital surveillance and CCTV developments include video analytics nowadays, to increase the functionality and effectiveness of both CCTV and access control systems, while solving the inherent difficulties caused by the sheer size of airports and their perimeters. CCTV systems are becoming increasingly interconnected and interdependent with other airport information systems, introducing additional vectors of attack, due to their interconnectivity. Thus, CCTV systems can be exposed to similar vulnerabilities as computers and networked devices. Specifically, weak network security may allow attackers to open a backdoor and exploit software vulnerabilities, which can enable the attacker to gain unauthorized access, as presented in Figure 11. Malware could also be uploaded during patching and with the collaboration of compromised employees (i.e. insider threat). A successful attack on CCTV systems would then allow an attacker to monitor all physical airport infrastructures. 

Impact Evaluation: Compromised CCTV systems impact airport operation safety both in landside and airside areas, which affects all security parameters: confidentiality, integrity and availability of security systems. In addition, in case that system administrators had to wipe the infected systems and reinstall the CCTV software, it’s possible that a good deal of footage to be lost and the system will be rendered inoperable for a time, which creates a serious safety handicap.

Cascading effects: Once a malicious actor has gained control of any CCTV device, this could lead to catastrophic impacts on airports safety. After a hacker has gained control, he could use the camera for hostile reconnaissance, or inject his own video stream, or even he could use the device to pivot into other devices on the same network; all of which would cause serious problems in airport security and airside operations safety.

Mitigation Actions: The first mitigation measure is to avoid connecting any CCTV device directly to the Internet, since cameras or CCTV systems, which can be remotely accessible with port forwarding all inbound traffic, are quite vulnerable to malicious attacks. Effective measures are the use of VPN (Virtual Private Network), use of non-standard network ports, while enabling dual factor authentication controls, when using a remote access service. Change of default passwords in all devices is one of the basic precaution actions, along with disabling unused services and closed equipment communication ports. Also, it is vital to provide adequate physical security to remote devices with security guard patrols especially in the perimeter of the airport installations, which can be the Achilles heel (weak point) for airport’s safety.

Resilience Measures: It is important to provide redundancies, by keeping legacy systems in standby mode, being activated when needed to support any relevant incident. Efficient security training to all airport employees could make difficult any attempt to interfere with CCTV systems, while incident response capabilities for security staff should be developed and regularly tested. Finally, an effective contingency plan should be developed, implemented and tested to improve airports resilience. 

#### 5.2.6. Malicious Attack: Misuse of Authorization

**Figure 12:**
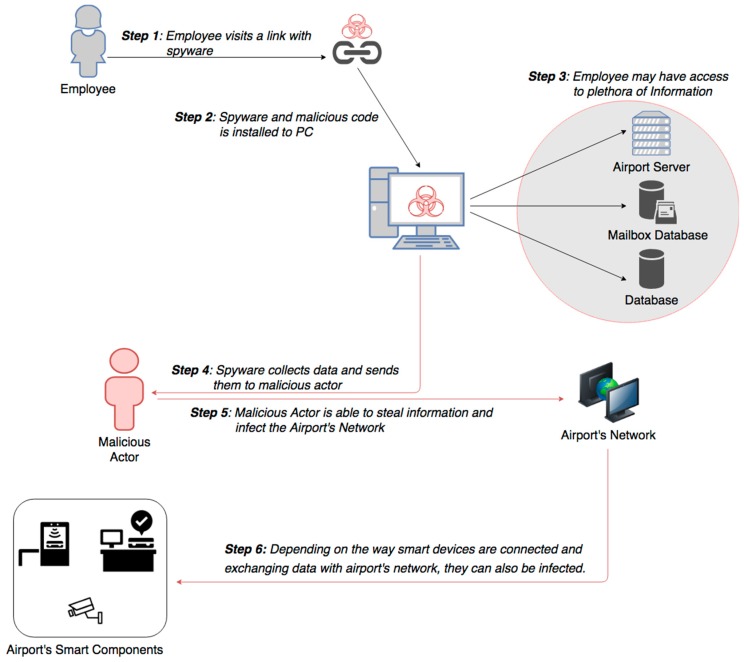
Misuse of authorization with APT.

Disgruntled employees, contractors or business associates having in possession access credentials may are able to misuse their authorization privileges and act as insider threat, aiming to steal information for personal gain or to benefit another organization. In addition, an intruder may gain access to airport’s network, using Advanced Persistent Threat (APT) as presented in Figure 12, remaining undetected for an extended period of time, while escalating authorization privileges. The intention of an APT attack is usually to monitor network activity and steal data rather than to cause damage to the network or organization. To maintain access to the targeted network without being discovered, threat actors use advanced methods, including continuously rewriting malicious code to avoid detection and other sophisticated evasion techniques [35]. Some APTs are so complex that they require full-time administrators to maintain and restore the compromised systems and software in the targeted network.

Impact Evaluation: Data which may be extracted are related to airport management and thus containing critical information for instance about airport vulnerabilities, airlines operation data or even passenger personal information. This can lead to large penalties, fines and loss of confidence. Data protection laws applicable worldwide, like the European General Data Protection Regulation (GDPR), provide severe penalties of up to 4% of company’s global turnover. Such an incident happened in 2018 with British Airways, where personal and financial data of approximately 380.000 customers were exposed. The airline admitted this data breach, while claiming that between those data weren’t any passport details or other sensitive personal data [36]. With compromised airport’s administration systems, there is a serious impact on operational safety both in landside and airside areas, which affects all security parameters: confidentiality, integrity and availability of airport’s essential services and operational systems. 

Cascading effects: Since the infected device may have access to plethora of information, privilege escalation from threat actors, who have breached their target systems, including gaining administrator rights, offer them the ability to move around the enterprise network at will [19]. Additionally, they can attempt to access other servers, as well as other secure areas of the network, facilitating their malicious indents and targets. Compromising airport’s systems puts at serious risk essential services and facilitates security attacks, causing civilian fatalities and serious financial losses.

Mitigation Actions: An effective user access management should be in place for granting and revoking access to all information systems and services. In addition, the use of utility programs that might be able to override system and application controls shall be restricted and tightly controlled. A variety of countermeasures are also necessary, including data encryption and antimalware, in order to mitigate such attack’s impacts [37]. Airport’s cybersecurity team should focus on detecting anomalies in outbound data to see if the network has been the target of any APT attack. 

Resilience Measures: The continuous monitoring and audit of systems and log files are crucial for the resilience of smart airports, since the data loss prevention is enhanced. Thus, any unauthorized action made by malicious insiders is immediately detected and appropriate actions are implemented. Moreover, airport’s employees and business associates should be granted the least level of privilege, and should be able to access only information that is needed, according to their working position and duties. Efficient security awareness and training to all airport employees could harden authorization misuse attempts, while incident response capabilities for security staff should be developed and regularly tested.

#### 5.2.7. Malicious Attack: Email Phishing and Social Engineering Attacks

**Figure 13:**
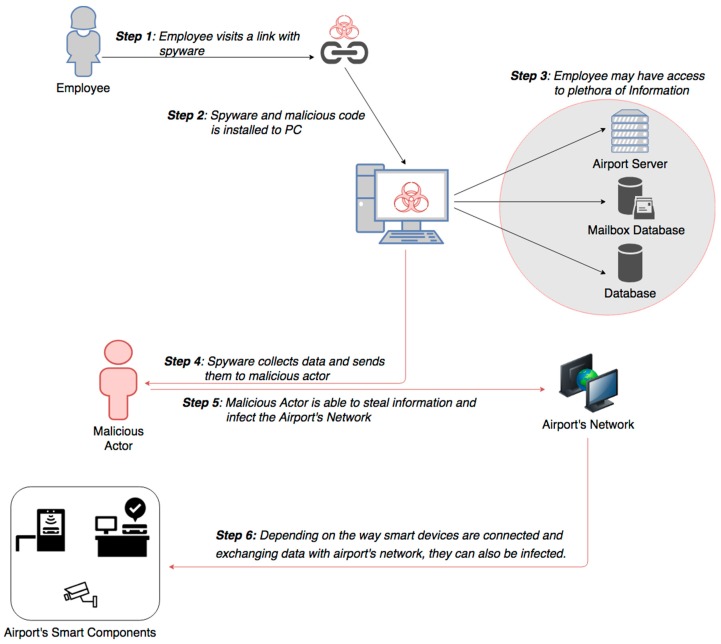
Social engineering attack scenario.

Social engineering, in the context of information security, refers to psychological manipulation of people into performing actions or divulging confidential information. It is a confidence trick for the purpose of information gathering, fraud, or system access [35]. Phishing is typically carried out by email spoofing or instant messaging, which often directs users to enter personal information at a fake website, identical to the legitimate one. Social Engineering and communications, purporting to be from social web sites, banks, online payment processors or IT administrators, are often used to lure victims. Such a scenario attack is presented in Figure 13. Even though organizations can install filtering capabilities, phishing emails still may get through, since phishers can use images instead of text to make it harder for anti-phishing filters to detect them. Hackers are spoofing email sender addresses from major companies and services, tricking recipients into thinking the malicious message is from a known source. Also, evil-twins is a phishing technique, where phisher creates a fake wireless network, that looks similar to a legitimate public network. This can be found in an airport and whenever someone logs on to the bogus network, fraudsters try to capture passwords and/or credit card information [35]. 

Impact Evaluation: Depending on the network privileges of IT devices all security factors can be serious impacted such as confidentiality, integrity and availability of airport operations. The degree of harm phishing attack is likely to cause is dependent on attacker’s motivation and victim’s organizational position and access privileges. A recent cyber-attack example, where ransom was attacker’s motive, happened in September 2018, at Bristol Airport. As a result, the airport’s information screens failed to operate for two days [38].

Cascading effects: Once airport’s authorization clearances and user access systems are compromised, the attacker depending on his motives can penetrate and attack other connected systems. Data alteration aims at whatever malicious act can compromise airport administration systems, where Airside and Landside operations may be infected causing passenger delays, cancelled flights and finally can jeopardize civilian’s safety, including potential terrorist facilitation. 

Mitigation Actions: The mitigation actions require a combination of technological, process, and people-based approaches. These actions include anti-spoofing control, filtering, dual authentication, malware protection and other technical security measures. In addition, user training, security awareness, encouraging employees to “Think before clicking a link” and being suspicious regarding emails that look strange or very attractive, including invitations from social media. Email addresses should be carefully examined and filtrated, while IT security team should be notified accordingly. It is obvious that many infiltration attempts could be stopped as a result of recipients being suspicious, mistrustful and paying more attention.

Resilience Measures: Phishing and social attacks work by exploiting weaknesses in human psychology and organizational culture. With security awareness and employees’ training, it is feasible to create an environment, which empowers users to report incidents for helping and increase the reporting rate of suspicious emails. These incidents can be further investigated with the use of virtual environments and sterile sandboxes, so as to inform and alert all airport stakeholders, as well as increase systems cyber resilience.

### 5.3. Smart Airport’s Malicious Attack Scenarios Synopsis

In order to facilitate airports stakeholders and cyber-security researchers to aggregate information exhibited in these cyber-attack scenarios, we have condensed information into a synoptic table. Table 1 summarizes this collection of malicious attack scenarios, presenting for each malicious threat which categories of assets may be affected, cascading effects, along with mitigation actions and resilience measures that can be taken, in order to improve airports cyber security and cyber resilience.

### 5.4. Malicious Attacks Motives

In this section, we introduce the attacker’s motivation into our scenario analysis, since people, while performing a malicious action, may fall on a subjective spectrum of good and evil. According to Safe Skies research [25] motives of cyber attackers fall into four general categories, all of which can reduce airport’s operational efficiency, as listed below:

(1) Political or Military: Foreign military or intelligence-related sources have the competences to conduct the most serious and harmful attacks. Their purpose is to gain some military, political, or strategic insight, affecting confidentiality, integrity and availability of systems to undermine public trust. Airports are highly symbolic and attractive targets for such attacks, where any disruption, impacts confidence in air traveling and national airspace safety.
(2) Commercial Espionage: Attackers with commercial espionage motivations are usually aligned to steal or damage confidential or proprietary information in order to gain commercial intelligence from private and public companies. This kind of attack aims to defraud, blackmail, obtain financial gains or targets corporate strategic goals. Examples of such commercial espionage targets are airports’ administration documents, including planning, construction, budget, financial, legal and government-related documents.
(3) Peer Group Disruption: Peer groups such as vandals, activists, or outsiders, along with a wide variety of individuals may engage in cyber-attacks, in order to disrupt or disable access to resources. They usually claim to have political reasons such as to protest, create economic harm, or rise status within their peer group. Distributed denial-of-service attacks are common examples in the airport environment, where attackers strive to prevent access to airport’s website or disrupt online services.
(4) Cybercrime: Attackers usually target networks and systems directly for data, in order to steal and resell valuable data, such as customer identification, credit card, or banking information. This is one of the most rapidly growing areas of attacks nowadays [25]. Airports that handle credit card information for paying services, such as baggage fees or parking allotments could be prime targets for these attackers. Although these attacks may be less sophisticated than the other types, cybercrime techniques and tools have been recently improved and become easier to obtain and use. For example, by using ransomware or destructive malware, attackers are able to encrypt or even destroy data and afterwards threaten their victims to pay a great amount of ransom (usually in bitcoins), in order to unlock data or refrain from exposing sensitive information.

Table 2 attributes to malicious threat scenarios discussed, the cybersecurity impact on assets according to the CIA triad (Confidentiality-Integrity-Availability) and the categories of cyber attackers who may be evolved in such malicious actions, according to their motives. 

## 6. Conclusions 

In this article, we have outlined how technological advances and IoT technologies may change the security threat models in aviation and influence the operational efficiency of smart airports. In order to extract information from airport security professionals, about their cyber-security efforts and risk management activities, we started our research with an online survey for airport’s cybersecurity. However, we confronted a decline from participants to fully complete the survey and provide detailed information about their cybersecurity implemented practices. This was a survey limitation, therefore, online desktop research for each airport’s technological situation and related work on cybersecurity best practices have also been investigated.

This study focused on cyber-attacks that may occur from malicious actions as the incorporation of smart applications in airports introduces new vulnerabilities. With the motive to increase cyber security awareness to all airport’s stakeholders, we have tried to expose in a simple and understandable way, key issues of cyber security in smart airports.

Commercial airports are required to develop their own policies to enhance cyber security, nowadays, since our survey has revealed that there is a large variation in the way airports implement measures to protect networked infrastructures and design cybersecurity solutions. Due to the fact that each airport has a variety of ICT applications, being operated within the airport perimeter, the resulting cyber security landscape has become very large and complicated.

Our research also revealed the disparity amongst airports in the methods and the degree of applying cyber security best practices. While smart airports are having a more mature cyber security posture, basic airports seem to have limited resources dedicated to cyber defense and resilience. Technical based cybersecurity practices have a better implementation rate for all airport categories, while organizational practices, policies and standards keep lower levels of implementation, including low levels of cyber security awareness and training prioritization.

Although smart airports perform the majority of good practices examined in our survey, security gaps have been revealed. Besides, the rapid advance of IoT technologies, along with the slower pace of the required regulatory processes, may lead to serious legal gaps for confronting malicious threats in smart airports. These gaps might pose challenges to smart airports for addressing security and safety. 

Ultimately, as our survey revealed, main security concern to all responders was security awareness. To this conclusion adds the fact that most malicious attacks are launched, due to untrained personnel in security issues. Therefore, the above analysis of attack scenarios, based on malicious intentions, can be supportive to airport community and aviation stakeholders to understand the meaning of acting proactively by implementing best cybersecurity practices. 

There is a need for identification and development of airport trust framework, helping operators navigate their trust relationships and indicate how smart devices and operators exchange data and operate together. Another important finding of our research was the growing need of educating IT experts and providing specialized advanced training in cybersecurity areas, in order to increase cybersecurity preparedness. Moreover, promoting security awareness of passengers and airports’ personnel on the risks posed by new IoT technologies is essential.

Securing Smart airports, against evolving cyber threats, is a shared responsibility for all aviation stakeholders, including commercial airports, airlines, business associates and regulators. As a result, a collaborative cyber-resilience model, which defines the appropriate cyber security posture for airports, is quite important nowadays. Airport operators ought to prioritize cyber security initiatives, in order to ensure safety of operations for airlines, passengers and public in general. Cyber threats and related risks will continue to grow, along with technological developments, while the relationship between safety and security in the aviation context will become more interdependent.

## Figures and Tables

**Table 1:** Smart Airport’s malicious attack aggregate analysis.

Malicious Attacks for Smart Airports				
THREAT DESCRIPTION	Assets Infected	Cascading Effects	Mitigation Actions	Resilience Measures
**DDoS Attacks**	- Web Services - Network Services - ATM communication -Wireless communications -Mobile telephony	-Airline/Airside Operations -Landside Operations -Airport Interoperability -IT and Comms	-Intrusion Detection/Protection (IDS/IPS) -Security hardening of systems -Firewalls, network segmentation - Volumetric protection from ISP	-Provide incident response & contingency plan - Regularly exercise preparedness - Communicate anomalous activity to airport stakeholders
**Communication Attacks**	-Air Traffic Control (ATM) -Communications, Navigation and Surveillance -Global Positioning System -Geographic Information Systems (GIS)	-IT and Comms -Airside Operations -Management of flight operations	- Intrusion Detection/Protection -Anti spoofing Control -Strong user authentication - WAM for ATM systems - Law enforcement in case of incident - Data encryption	-Provide incident response capabilities for airports (including airlines) -Maintain Communication BackUp Alternatives fully operational
**Malicious Software**	-Network and IT systems -SCADA Systems -Staff smart devices -Passenger IT devices -Operational Servers	-Airline/Airside -Landside Operations -Passenger Management System -IT and Comms -Safety and Security	-Intrusion Detection/Protection (IDS/IPS) -Antimalware & technical control -BYOD controls -Least privilege access manag. -Software and hardware updates -Application security and secure design according to Inter.Stds	-Provide incident response & contingency plan -Develop forensic analytic capabilities - Regularly exercise preparedness and response time on test incidents - Security Awareness and Training
**Tampering with Airport Devices**	-Common Use Passenger Processing Systems -Baggage Handling -Passenger Ticketing System	-Local Area Network -Landside Operation Systems -Passenger Management	-Resctrict Usage of Ext. Devices - Intrusion Detection/Protection - Data Encryption - Enhance Physical Security and Surveillance systems	- Provide BackUp Alternatives - Monitor risk effectively - Regularly exercise incident response of airport staff - Implement Contigency Plan
**Network Attacks**	-ICS SCADA -CCTV systems -Baggage handling -Landside Operations	-Facilities and Maintenance -Airside Operations -Landside Operations -Baggage handling -IT and Comms	-Firewalls, network segmentation and defence in depth - Intrusion Detection/Protection -Strong user authentication -Change default administrator credentials of devices -BYOD controls -Data encryption	- Provide BackUp Alternatives - Monitor risk effectively -Develop forensic analytic capabilities - Provide incident response capabilities for airports - Implement Contigency Plan - Security Awareness and Training
**Misuse of Authorisation**	-SCADA systems -Air Traffic Management -Enterprise Management System -Access Control & Surveillance -IT Systems	-Facilities and Maintenance -Airport Administration -Airline/Airside Operations -Landside Operations	-Change default credentials of devices -BYOD controls -Software and hardware updates -Least privilege and data classification -Data encryption -Strong user authentication -User access management	-Provide incident response capabilities -Develop forensic analytic capabilities - Regularly exercise preparedness and response time on test incidents - Implement Contigency Plan - Security Awareness and Training
**Social and Phishing Attacks**	-Enterprise Management System -Landside Operations -SCADA Systems -IT and Comms	-Airport Administration -Airline/Airside Operations -Landside Operations -Facilities and Maintenance	- Intrusion Detection/Protection -Software and hardware updates -Firewalls, network segmentation and defence in depth -Anti spoofing Control -Strong user authentication -Application security and secure design	-Provide incident response capabilities -Create a culture of security - Develop forensic analytic capabilities - Encourage employees to “Think before you click” - Establish and populate corporate and ethics rules

**Table 2:** Malicious attack motives analysis.

Scenario No	Malicious Attack Scenario	Impact on			Malicious Attack Motives			
		Confidentiality	Integrity	Availability	Political or Military	Commercial Espionage	Peer Group Disruption	Cybercrime
1	Distributed Denial of Service attacks			**√**	**√**		**√**	**√**
2	Communication attack to ATM systems	**√**	**√**	**√**	**√**	**√**	**√**	**√**
3	Malicious Software on Airport’s Network	**√**	**√**	**√**	**√**	**√**		**√**
4	Tampering with airport self-serving systems	**√**	**√**	**√**	**√**		**√**	**√**
5	Network attack to CCTV systems	**√**	**√**	**√**	**√**	**√**	**√**	**√**
6	Misuse of Authorization		**√**	**√**	**√**		**√**	**√**
7	Email Phishing and Social Engineering Attacks	**√**	**√**	**√**	**√**	**√**		**√**

**Figure A1:**
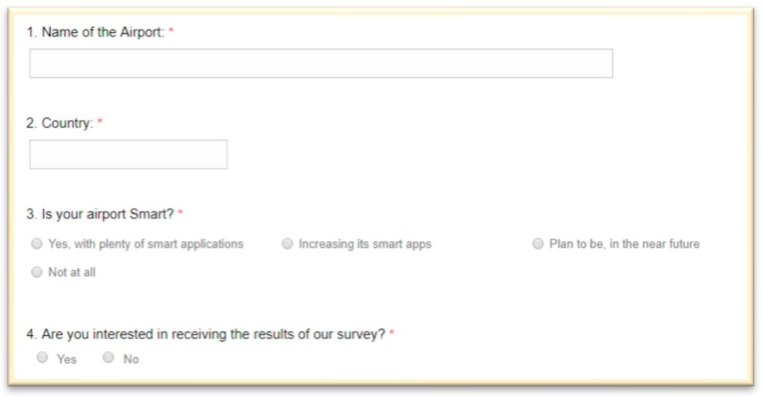
Questions about the airport’s details.

**Figure A2:**
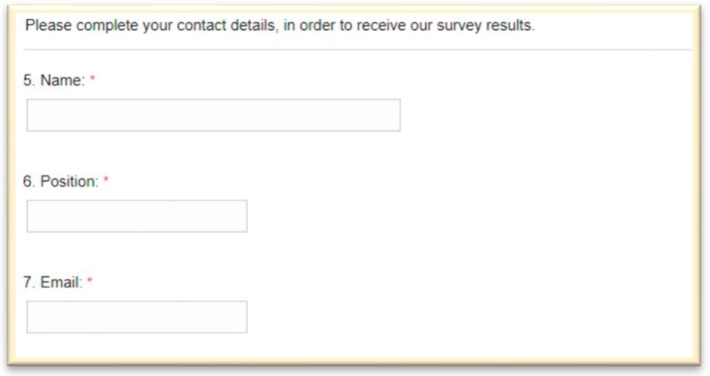
Questions for contact details.

**Figure A3:**
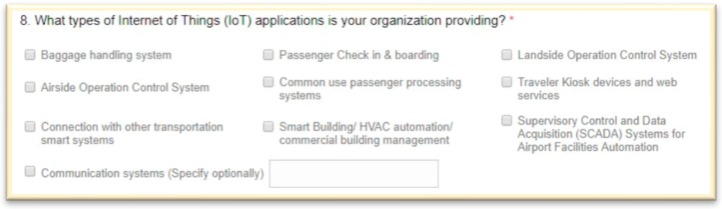
Question No. 8.

**Figure A4:**
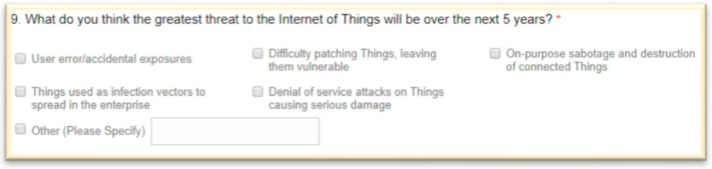
Question No. 9.

**Figure A5:**
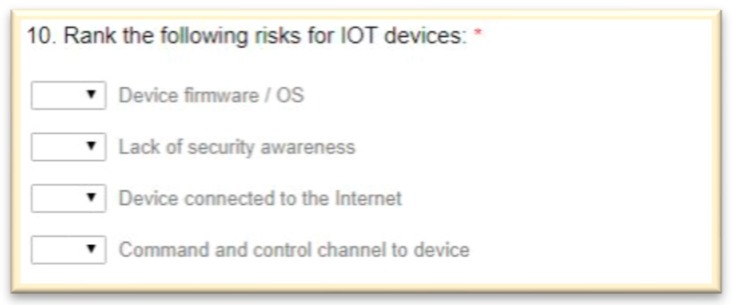
Question No. 10

**Figure A6:**
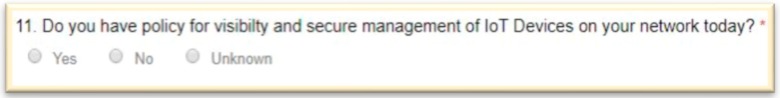
Question No. 11.

**Figure A7:**
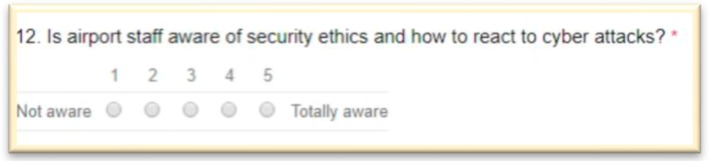
Question No. 12.

**Figure A8:**
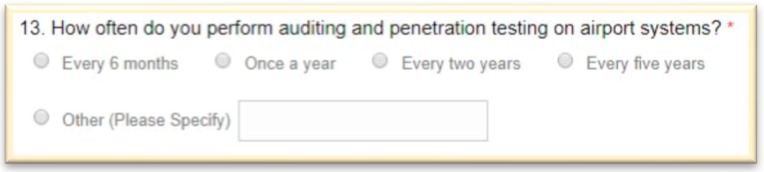
Question No. 13.

**Figure A9:**
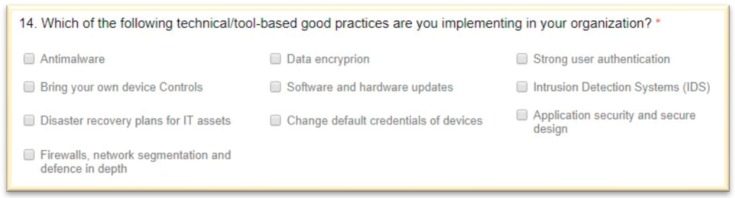
Question No. 14.

**Figure A10:**
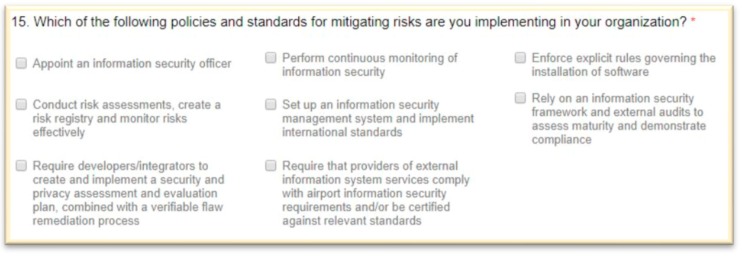
Question No. 15.

**Figure A11:**
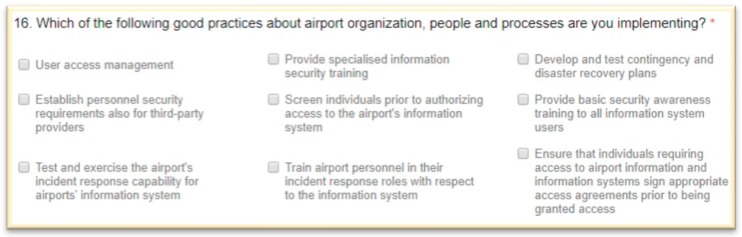
Question No. 16.

## Appendix A

This appendix presents the questionnaire addressed to Cyber Security in Smart Airports, which has been sent to the busiest 200 commercial European and American airports. This survey was addressed to both Smart airports or not. Its main purpose was to understand the opinion of Airport IT personnel about the introduction of the IoT technology to Airports and whether they have the appropriate security awareness. This online survey took place in 1^st^ semester of 2017 and invitation was sent through email to IT personnel.

Also, it is worth-mentioning that all survey responses that we received are strictly confidential and data from this research are reported only in the aggregate. Although, in this online survey we have counted 280 visits to our online questionnaire, we have finally received only 34 fully completed answers and we elaborated these results.

**Online Survey Questionnaire**

The questionnaire was available in the following link: https://survey.zohopublic.eu/zs/uiCChZ.

The questions were the following:

In case that the responder answers yes in question No. 4, then he should fill some contact details. Otherwise, the responder will continue answering the rest questions, starting with the Question No. 8 (Figure A3).

**Online Survey Questionnaire Answers and Related Statistics**

**Table A1:** Origin of airports’ answering & airport’s classification.

**Airport’s Location**	**Answers Received**	
Europe	66%	22
USA	34%	12
TOTAL	100%	34
**Airport’s Classification**	**Answers Received**	
Basic	16%	5
Agile	56%	19
Smart	28%	10
TOTAL	100%	34

**Table A2:** Answers to Question 8.

Question: What Types of Internet of Things (IoT) Applications is Your Organization Providing?		
IoT Applications in Airports	Answers Received	
SCADA	6%	2
Connection with other transportation systems	15%	5
Landside Operation Control System	18%	6
Airside Operation Control System	24%	8
Communication systems	27%	9
Baggage handling system	29%	10
Smart Building/HVAC/BMS	29%	10
Traveler Kiosk devices and web services	29%	10
Common use passenger processing systems	41%	14
Passenger Check in & boarding	41%	14

**Table A3:** Answers to Question 14.

Question: Which of the Following Technical/Tool-Based Good Practices are You Implementing in Your Organization?				
Technical Good Practices	BASIC	AGILE	SMART	ALL
Antimalware	2	10	6	18
Software and hardware updates	2	15	8	25
Firewalls & network segmentation	4	19	10	33
Intrusion Detection Systems	0	11	6	17
Strong user authentication	0	15	6	21
Change default credentials	2	10	4	16
Data encryption	0	10	8	18
BYOD Controls	0	6	4	10
Disaster recovery plans	2	11	10	23
Appl. security & secure design	2	6	8	16

**Table A4:** Answers to Question 16.

Question: Which of the Following Policies and Standards for Mitigating Risks are You Implementing in Your Organization?				
Good Practices for Policies and Standards	BASIC	AGILE	SMART	ALL
User access management	2	10	10	22
Screen individuals prior to authorize access to airport’s IT system	1	6	6	13
Ensure access agreement to individuals prior to grant access	2	10	8	20
Personnel security requirements for third-party providers	2	8	6	16
Basic security awareness training to all information system users	2	10	8	20

**Table A5:** Answers to Question 15.

Question: Which of the Following Policies and Standards for Mitigating Risks Are You Implementing in Your Organization?				
Good Practices About People, Organization and Processes	BASIC	AGILE	SMART	ALL
User access management	5	17	10	32
Screen individuals prior to authorize access to airport’s IT system	0	10	6	16
Ensure access agreement to individuals prior to grant access	2	4	4	10
Personnel security requirements for third-party providers	2	6	6	14
Basic security awareness training to all information system users	2	10	8	20
Specialised info security training	2	6	4	12
Train airport personnel in incident response for IT system	2	6	4	12
Test and exercise incident response capability for IT system	2	10	6	18
